# PPP/INS Tight Integration with BDS−3 PPP−B2b Service in the Urban Environment

**DOI:** 10.3390/s23052652

**Published:** 2023-02-28

**Authors:** Luguang Lai, Xin Meng, Dongqing Zhao, Xin Li, Wenzhuo Guo, Linyang Li

**Affiliations:** 1 Institute of Surveying and Mapping, Information Engineering University, Zhengzhou 450001, China; 2 College of Computer and Information, Hohai University, Nanjing 211100, China; 3 School of Geodesy and Geomatics, Wuhan University, Wuhan 430079, China

**Keywords:** BDS−3, PPP−B2b, urban scenarios, real−time PPP/INS, MEMS IMU

## Abstract

To provide continuous and reliable real−time precise positioning services in challenging environments and poor internet conditions, the real−time precise corrections of the BeiDou global navigation satellite system (BDS−3) PPP−B2b signal are utilized to correct the satellite orbit errors and clock offsets. In addition to this, using the complementary characteristics of the inertial navigation system (INS) and the global navigation satellite system (GNSS), a PPP−B2b/INS tight integration model is established. With observation data collected in an urban environment, the results show that PPP−B2b/INS tight integration can ensure a decimeter−level positioning accuracy; the positioning accuracies of the E, N, and U components are 0.292, 0.115, and 0.155 m, respectively, which can provide a continuous and secure position during short interruptions in the GNSS. However, there is still a gap of about 1 dm compared with the three−dimensional (3D) positioning accuracy obtained from Deutsche GeoForschungsZentrum (GFZ) real−time products, and a gap of about 2 dm compared with the GFZ post−precise products. Using a tactical inertial measurement unit (IMU), the velocimetry accuracies of the tightly integrated PPP−B2b/INS in the E, N, and U components are all about 0.3 cm/s, and the attitude accuracy of yaw is about 0.1 deg, while the pitch and roll show a superior performance of less than 0.01 deg. The accuracies of the velocity and attitude mainly depend on the performance of the IMU in the tight integration mode, and there is no significant difference between using real−time products and post products. The performance of the microelectromechanical system (MEMS) IMU and tactical IMU is also compared, and the positioning, velocimetry, and attitude determinations with the MEMS IMU are significantly worsened.

## 1. Introduction

With the rapid development of automatic driving, the Internet of Things, robots, and other fields, greater requirements for the timeliness and reliability of precise positioning have been put forward [1]. As a vital infrastructure, the global navigation satellite system (GNSS) is extensively used in both civil and military domains. Presently, precise point positioning (PPP) and real−time kinematic (RTK) positioning are widely used in the GNSS field. RTK can provide a real−time (RT) centimeter−level position, but it requires the prior deployment of reference stations, lacks flexibility, and brings a large communication burden [2]. Using a single receiver, PPP can meet global kinematic decimeter−level and static centimeter−level positioning. However, traditional PPP is usually post−solution due to the long latency of precise products [3,4]. With the rapid development of RT products, RT−PPP has turned into a reality. However, the positioning accuracy of RT−PPP depends on the accuracy of external satellite orbit and clock offset products.

Currently, the acquisition of RT−PPP corrections can be divided into two methods: one is from internet communication and the other is based on satellite communication [5]. The former is mainly implemented by the real−time service (RTS), which has been officially provided by the International GNSS Service (IGS) since 2013, and users can freely download the RT state−space representation (SSR) corrections via the internet and the broadcast ephemeris is corrected thereafter [6]. Evaluation results show that the accuracy of the RT orbit and clock offsets from the IGS RTS are better than 5 cm and 0.15 ns, respectively [7]. Although the IGS RTS product can be freely obtained through the internet, its dependence on network communication limits its service coverage, especially for areas without network communication coverage such as the ocean and desert. In addition, users cannot obtain RTS products in the case of network congestion and a poor network signal, resulting in an interruption of RT−PPP. In the second method, satellite communication can overcome the aforementioned disadvantages, and typical representatives are the commercially available RT−PPP systems offered by some companies [8,9]. RT−PPP based on satellite communication is relatively more mature, but it requires special receivers and is slightly more expensive. However, with the incorporation of RT orbit and clock corrections by the BeiDou global navigation satellite system (BDS−3), Galileo, and other satellite systems, RT−PPP can be feasibly and effectively obtained nowadays [10].

Among these satellite navigation systems, the BDS−3 can provide RT−PPP services for China and the surrounding regions by broadcasting PPP−B2b signals via three geostationary earth orbit (GEO) satellites, which currently only support the global positioning system (GPS) and BDS−3; these services will incorporate Galileo and GLONASS satellite systems in the near future [11]. Currently, the clock offset precision of the PPP−B2b service is approximately 0.2 ns, which is slightly lower than the accuracy of the Centre National d’Etudes Spatiales (CNES) products [5]. Many scholars have shown that the PPP−B2b service can achieve centimeter− and decimeter−level positioning in static and kinematic scenarios, respectively, in China and surrounding areas [12,13,14,15]. However, static stations with high−performance receivers in open scenes were utilized, dynamic positioning was also performed in a simulated approach, and the positioning performance in real dynamic scenes is unknown.

For RT−PPP in real dynamic scenes, ref. [16] achieved a vehicle−mounted positioning accuracy of about 0.2 m in open scenes using RTS products. Ref. [17] carried out an ocean RT−PPP experiment using the PPP−B2b service, and a three−dimensional (3D) positioning accuracy of 18.2 cm was achieved. A dynamic vehicle−mounted experiment was carried out in ref. [18] and the positioning errors exceeded 1 m due to environmental occlusion. However, in challenging environments, GNSS signals are highly susceptible to blocking and the multi−path effect is obvious. Furthermore, in complicated scenarios, such as boulevards, urban canyons, overpasses, etc., GNSS signals are easily interrupted; hence, continuous and reliable precise positioning cannot be achieved only by using GNSS, and other sensors must be integrated to assure that a continuous and reliable position is provided [19,20]. The inertial navigation system (INS) has the advantages of independence and a precise short−term resolution, indicating great complementary features with the GNSS [21,22,23,24]. The RT positioning performance of a tight integration using single−frequency BDS, GPS, and INS was analyzed with RTS products, and a sub−meter level positioning accuracy was achieved in a sheltered campus environment [25]. Ref. [26] implemented a GPS and GLONASS single−frequency PPP/INS loose integration using CNES RT corrections and added motion constraints to the PPP/INS system; its positioning accuracy still exceeded 1 m in a complex urban environment. Ref. [27] performed an RT PPP/INS tight combination in the urban environment using SSR corrections from different analysis centers, and its 3D positioning error was still around 1 m. Ref. [28] investigated the performance of a loosely integrated BDS−3 PPP−B2b/INS, and the positioning accuracy was about 0.36 m in an open environment and about 0.85 m in a sheltered environment. However, the accuracy of loosely integrated PPP−B2b/INS was not compared with other precise products, the performance of the velocimetry and attitude determination were not considered, and only the loose integration mode was realized. Compared with loose integration, the PPP/INS tight integration takes full advantage of the accurate short−term navigation resolution of the INS and hence features robust quality control and verification, contributing to resisting GNSS gross error, cycle slip detection, and reconvergence after GNSS outages [29,30,31]. Therefore, the existing research into BDS−3 PPP−B2b/INS is rather insufficient, especially in terms of the PPP−B2b/INS tight integration. In this paper, we proposed a tightly integrated BDS−3 PPP−B2b/INS model, its performance of on−board experiments in the urban environment was analyzed, and the results using the RT and post products from Deutsche GeoForschungsZentrum (GFZ) were compared. In addition, the performance of the microelectromechanical system (MEMS) inertial measurement unit (IMU), which is preferred in mass applications due to its low price, light weight, compact volume, and low−energy consumption, was further evaluated.

The rest of the paper is structured as follows: In Section 2, the methodology of the tightly integrated PPP−B2b/INS is briefly introduced. In Section 3, the origins and processing methods of the on−board experiment are presented, the performance of the tightly integrated PPP−B2b/INS is evaluated, and the results of the low−cost MEMS IMU are also detailed. The conclusions and perspectives are discussed in Section 4.

## 2. Methodology

In this section, we first introduce the calculation of PPP−B2b precise products, then we present the real−time ionosphere−free PPP model using the PPP−B2b service, and finally, the tightly integrated PPP−B2b/INS is presented.

### 2.1. Precise Products Calculation with PPP−B2b Service

The broadcasted PPP−B2b correction information is based on the BeiDou Time (BDT) and BeiDou coordinate system (BDCS) [32], and the update interval for satellite orbit corrections and clock offset corrections are 48 s and 6 s, respectively. The broadcasted orbit corrections, $\delta X_{B2b}$, are components of radial, along−track, and cross−track directions under the satellite fixed coordinate system, while the satellite position calculated by the broadcast ephemeris is under the earth−centered, earth−fixed (ECEF) frame. Therefore, the corrections can be converted to the ECEF frame as follows:

(1) $$\delta X^{s}=\left[\begin{array}{ccc} e_{radial} & e_{along} & e_{cross} \end{array}\right]\cdot \delta X_{B2b}$$

with

(2) $$\left\{ \begin{array}{l} e_{radial}=\frac{r}{\left|r\right|}\\ e_{cross}=\frac{r\times \dot{r}}{\left|r\times \dot{r}\right|}\\ e_{along}=e_{cross}\times e_{radial} \end{array}\right.$$

where $\delta X^{s}={\left[\begin{array}{ccc} \delta x & \delta y & \delta z \end{array}\right]}^{T}$ is the orbit correction vector derived from PPP−B2b for satellite s in the ECEF frame and r and $\dot{r}$ represent the satellite position and velocity vectors in the ECEF frame, respectively.

Then, the precise satellite orbit, $X_{prec}^{s}$, can be obtained as follows:

(3) $$X_{prec}^{s}=X_{brdc}^{s}-\delta X^{s}$$

where $X_{brdc}^{s}$ is the satellite position calculated using broadcast ephemeris.

In contrast, the satellite clock offsets correction, $C_{0}$, from PPP−B2b can be directly used to correct the broadcast clock offsets, $\delta t_{brdc}^{s}$, to obtain RT precise clock offsets, $\delta t_{prec}^{s}$:

(4) $$\delta t_{prec}^{s}=\delta t_{brdc}^{s}-\frac{C_{0}}{c}$$

where c denotes the speed of light in a vacuum.

Currently, the PPP−B2b service only supports different code biases (DCB) products of the BDS−3 with an update interval of 48 s. Multiple BDS−3 signals, including B1I, B1C, B2a, B2b, and B3I, are broadcasted, among which the clock offsets reference of the BDS−3 B1C broadcast ephemeris is the B3I signal, and hence multiple types of DCB corrections are provided.

### 2.2. Real−Time Ionosphere−Free PPP Model

The GNSS code, $P_{r,i}^{s}$, and carrier phase, $L_{r,i}^{s}$, measurement equations between station r and satellite s at frequency i can be described in the following form [33]:

(5) $$\begin{array}{l} P_{r,i}^{s}=\rho_{r}^{s}+t_{r}-t^{s}+T_{r}^{s}+\gamma_{i}\cdot I_{r,1}^{s}+d_{r,i}-d_{i}^{s}+\varepsilon_{r,i}^{s}\\ L_{r,i}^{s}=\rho_{r}^{s}+t_{r}-t^{s}+T_{r}^{s}-\gamma_{i}\cdot I_{r,1}^{s}+\lambda_{i}\left(N_{r,i}^{s}+b_{r,i}-b_{i}^{s}\right)+\xi_{r,i}^{s} \end{array}$$

where $\rho_{r}^{s}$ denotes the geometry distance between the satellite and receiver; $t^{s}$ and $t_{r}$ represent the satellite and receiver clock offsets, respectively; $T_{r}^{s}$ is the tropospheric delay; $I_{r,1}^{s}$ is the ionospheric propagation delay at the first frequency; $\lambda_{i}$ represents the wavelength; $\gamma_{i}=f_{1}^{2}/f_{i}^{2}$ denotes the frequency−dependent amplification factor of the ionosphere; $N_{r,i}^{s}$ is the integer ambiguity; $d_{i}^{s}$ and $d_{r,i}$ are the code hardware delays for the satellite and receiver, respectively; $b_{i}^{s}$ and $b_{r,i}$ represent the satellite and receiver phase hardware delays, respectively; and $\varepsilon_{r,i}^{s}$ and $\xi_{r,i}^{s}$ denote the observation noise of the code and carrier phase, respectively. The other effects, such as the phase wind−up, tidal load deformation, relativistic effect, etc., have been corrected according to the empirical model [34].

The effect of first−order ionospheric delay is eliminated using the ionosphere−free (IF) combination model in this paper. For the GPS, the precise clock offsets recovered from Equation (4) can be used directly. However, the clock offsets reference of the BDS−3 broadcast ephemeris is the B3I signal. The satellite clock offsets of the BDS−3 and GPS be expressed as [17]:

(6) $$\begin{array}{l} {\hat{t}}^{s,C}=t^{s}+d_{B3I}^{s,C}\\ {\hat{t}}^{s,G}=t^{s}+d_{IF}^{s,G} \end{array}$$

with

(7) $$\left\{ \begin{array}{l} d_{IF}^{s,G}=\alpha \cdot d_{i}^{s}+\beta \cdot d_{j}^{s}\\ \alpha =\;f_{i}^{2}/\left(f_{i}^{2}-f_{j}^{2}\right)\\ \beta =-f_{j}^{2}/\left(f_{i}^{2}-f_{j}^{2}\right) \end{array}\right.$$

where the superscript C and G represent the BDS−3 and GPS, respectively; ${\hat{t}}^{s,C}$ and ${\hat{t}}^{s,G}$ denote the BDS−3 and GPS IF satellite clock offsets, respectively; $d_{B3I}^{s,C}$ represents the code hardware delays of the B3I signal at the satellite side; and α and β are the IF combination factors.

For BDS−3, B1I and B3I signals are used in this paper. The recovered clock offsets from Equation (4) contain the B3I signal satellite code delays. Hence, DCB products are needed for BDS−3 satellites. The corresponding observation equations are:

(8) $$\left\{ \begin{array}{l} {\tilde{P}}_{r,IF}^{s,C}=\rho_{r}^{s,C}+{\hat{t}}_{r,IF}-{\hat{t}}^{s,C}+T_{r}^{s,C}+\varepsilon_{r,IF}^{s,C}\\ L_{r,IF}^{s,C}=\rho_{r}^{s,C}+{\hat{t}}_{r,IF}-{\hat{t}}^{s,C}+T_{r}^{s,C}+\lambda_{IF}^{C}{\hat{N}}_{r,IF}^{s,C}+\xi_{r,IF}^{s,C}\\ P_{r,IF}^{s,G}=\rho_{r}^{s,G}+{\hat{t}}_{r,IF}-{\hat{t}}^{s,G}+T_{r}^{s,G}+ISB^{G-C}+\varepsilon_{r,IF}^{s,G}\\ L_{r,IF}^{s,G}=\rho_{r}^{s,G}+{\hat{t}}_{r,IF}-{\hat{t}}^{s,G}+T_{r}^{s,G}+\lambda_{IF}^{G}{\hat{N}}_{r,IF}^{s,G}+ISB^{G-C}+\xi_{r,IF}^{s,G} \end{array}\right.$$

with

(9) $$\left\{ \begin{array}{l} {\tilde{P}}_{r,IF}^{s,C}=\alpha \cdot \left(P_{r,B1I}^{s,C}+DCB_{B1I-B3I}^{s,C}\right)+\beta \cdot P_{r,B3I}^{s,C}\\ N_{r,IF}^{s,sys}=\left(\alpha \cdot \lambda_{i}^{sys}N_{r,i}^{s,sys}+\beta \cdot \lambda_{j}^{sys}N_{r,j}^{s,sys}\right)/\lambda_{IF}^{sys}\\ {\hat{N}}_{r,IF}^{s,C}=N_{r,IF}^{s,C}+\left(b_{r,IF}^{C}-b_{IF}^{s,C}-d_{r,IF}^{C}+d_{B3I}^{s,C}\right)/\lambda_{IF}^{C}\\ {\hat{N}}_{r,IF}^{s,G}=N_{r,IF}^{s,G}+\left(b_{r,IF}^{G}-b_{IF}^{s,G}-d_{r,IF}^{G}+d_{IF}^{s,G}\right)/\lambda_{IF}^{G} \end{array}\right.$$

where the script sys represents the satellite system and $DCB_{B1I-B3I}^{s,C}$ represents the DCB between the B1I and B3I signal. Since the receiver clock offsets between different satellite systems are not consistent, an additional inter−system bias, $ISB^{G-C}$, needs to be introduced, and the satellite systems used in this paper include the BDS−3 and GPS.

When the precise satellite orbits and clock offsets recovered by PPP−B2b are adopted, the errors of the satellite orbits and clocks are no longer considered and the linearized IF observation equation can be expressed as:

(10) $$\begin{array}{l} p_{r,IF}^{s,sys}=n_{r}^{s,sys}\cdot \delta x+{\hat{t}}_{r,IF}+m_{r,w}^{s,sys}\cdot Z_{r,w}+ISB_{G-C}+\varepsilon_{r,IF}^{s,sys}\\ l_{r,IF}^{s,sys}=n_{r}^{s,sys}\cdot \delta x+{\hat{t}}_{r,IF}+m_{r,w}^{s,sys}\cdot Z_{r,w}+\lambda_{IF}^{sys}{\hat{N}}_{r,IF}^{s,sys}+ISB_{G-C}+\xi_{r,IF}^{s,sys} \end{array}$$

where $p_{r,IF}^{s,sys}$ and $l_{r,IF}^{s,sys}$ represent observed−minus−computed (OMC) observations of the code and phase, respectively; $n_{r}^{s,sys}$ indicates the unit vector from the station to the satellite; δx is the vector of three−dimensional position corrections; $Z_{r,w}$ is the zenith wet delay; and $m_{r,w}^{s,sys}$ is the mapping function. The dry component of tropospheric delays can be corrected accurately using empirical models [35], whereas the wet component is calculated in the procedure.

### 2.3. Tightly Integrated Model of PPP−B2b/INS

The error equation of the strapdown INS is the basis for the discussion of the INS error propagation law, initial alignment, and integration navigation. In this paper, the INS error equations expressed in the ECEF frame are directly provided [30]:

(11) $$\left[\begin{array}{l} \delta {\dot{r}}^{e}\\ \delta {\dot{v}}^{e}\\ {\dot{\varphi }}^{e} \end{array}\right]=\left[\begin{array}{c} \delta v^{e}\\ -2\Omega_{ie}^{e}\delta v^{e}+\left[(C_{b}^{e}f_{ib}^{b})\times \right]\varphi^{e}+C_{b}^{e}\delta f_{ib}^{b}\\ -\Omega_{ie}^{e}\varphi^{e}-C_{b}^{e}\delta \omega_{ib}^{b} \end{array}\right]$$

where the scripts i, b, and e represent earth−centered inertial, body, and ECEF frames, respectively; $\delta r^{e}$, $\delta v^{e}$, and $\varphi^{e}$ are the position, velocity, and misalignment error expressed in the ECEF frame with the differential values of $\delta {\dot{r}}^{e}$, $\delta {\dot{v}}^{e}$, and ${\dot{\varphi }}^{e}$, respectively; $f_{ib}^{b}$ denotes the specific force obtained from the accelerometer; $\Omega_{ie}^{e}$ denotes the skew−symmetric form of the earth rotation rates $\omega_{ie}^{e}$; $C_{b}^{e}$ represents the rotation matrix from frame b to frame e; $\omega_{ib}^{b}$ is the angular rate obtained from the gyroscope; and $\delta \omega_{ib}^{b}$ and $\delta f_{ib}^{b}$ are the synthetic errors of the gyroscope and accelerometer, respectively. In this paper, only the biases are considered, which are further modeled as a random walk process.

The INS state model is expressed in the following form:

(12) $$\underset{{\dot{X}}_{INS}}{\underset{︸}{\left[\begin{array}{l} \delta {\dot{r}}^{e}\\ \delta {\dot{v}}^{e}\\ {\dot{\varphi }}^{e}\\ \delta {\dot{b}}_{a}\\ \delta {\dot{b}}_{g} \end{array}\right]}}=\underset{F_{INS}}{\underset{︸}{\left[\begin{array}{ccccc} 0 & I & 0 & 0 & 0\\ 0 & -2\omega_{ie}^{e}\times & f^{e}\times & C_{b}^{e} & 0\\ 0 & 0 & -\omega_{ie}^{e}\times & 0 & -C_{b}^{e}\\ 0 & 0 & 0 & 0 & 0\\ 0 & 0 & 0 & 0 & 0 \end{array}\right]}}\underset{X_{INS}}{\underset{︸}{\left[\begin{array}{l} \delta r^{e}\\ \delta v^{e}\\ \varphi^{e}\\ \delta b_{a}\\ \delta b_{g} \end{array}\right]}}+\underset{w_{INS}}{\underset{︸}{\left[\begin{array}{c} \xi_{r}\\ \xi_{v}\\ \xi_{\varphi }\\ \xi_{a}\\ \xi_{g} \end{array}\right]}}$$

where $\delta b_{a}$ and $\delta b_{g}$ are the biases of the accelerometer and gyro, respectively, and ξ represents the process noise of the corresponding parameters.

The PPP−B2b/INS tight integration model is based on the original observation data and its state equation is the fusion of the GNSS− and INS−related state terms, where the GNSS−related state equation is:

(13) $$\underset{{\dot{X}}_{GNSS}}{\underset{︸}{\left[\begin{array}{l} \delta {\dot{\hat{t}}}_{r,IF}\\ \delta I\dot{S}B\\ \delta {\dot{Z}}_{r,w}\\ \delta {\dot{\hat{N}}}_{r,IF}^{s} \end{array}\right]}}=\underset{F_{GNSS}}{\underset{︸}{\left[\begin{array}{cccc} 0 & 0 & 0 & 0\\ 0 & 0 & 0 & 0\\ 0 & 0 & 0 & 0\\ 0 & 0 & 0 & 0 \end{array}\right]}}\underset{X_{GNSS}}{\underset{︸}{\left[\begin{array}{l} \delta {\hat{t}}_{r,IF}\\ \delta ISB\\ \delta Z_{r,w}\\ \delta {\hat{N}}_{r,IF}^{s} \end{array}\right]}}+\underset{w_{INS}}{\underset{︸}{\left[\begin{array}{l} 0\\ \xi_{ISB}\\ \xi_{Z}\\ 0 \end{array}\right]}}$$

The state equation of the tightly integrated PPP−B2b/INS can be described as:

(14) $$\left[\begin{array}{l} {\dot{X}}_{INS}\\ {\dot{X}}_{GNSS} \end{array}\right]=\left[\begin{array}{cc} \begin{array}{c} F_{INS}\\ 0 \end{array} & \begin{array}{c} 0\\ F_{GNSS} \end{array} \end{array}\right]\left[\begin{array}{l} X_{INS}\\ X_{GNSS} \end{array}\right]+\left[\begin{array}{l} w_{INS}\\ w_{GNSS} \end{array}\right]$$

The observation equations of the tightly integrated PPP−B2b/INS are built on the basis of IF PPP Equation (10), while the INS−predicted observations obtained from the INS−mechanized results are utilized as computed values. In addition, the spatial lever arm error must be considered in advance when integrating IMU and GNSS observations due to the inconsistency between the IMU center and the antenna phase center.

**Figure 1:**
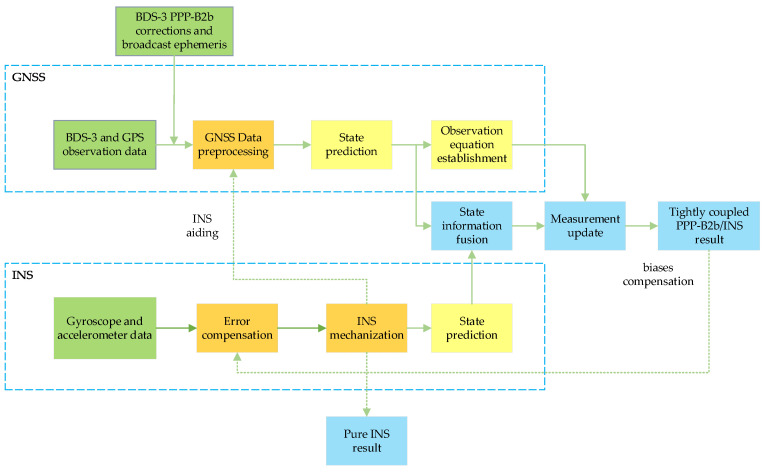
Flowchart of tightly integrated PPP−B2b/INS.

The flowchart of tightly integrated PPP−B2b/INS is shown in Figure 1, which contains three parts: GNSS, INS, and integration. Using the observation data, BDS−3 PPP−B2b corrections, and the broadcasted ephemeris, the GNSS unit primarily contains data pre−processing, such as gross error and cycle slip detection, GNSS state prediction, and PPP equation construction. An initial alignment of the system is conducted with the position provided by the GNSS in the INS unit; the observation data obtained from the accelerometer and gyroscope are then used for mechanization to calculate the present INS navigation information which can contribute to the pre−processing of the GNSS. Then, the state parameters from the INS and GNSS are integrated and updated using the GNSS observations to gain the tightly integrated PPP−B2b/INS solution. The IMU biases will be fed back to the subsequent IMU data to restrict the INS error accumulation. The PPP−B2b/INS tight integration model will output a pure INS result from INS mechanization when the GNSS signals are lost.

## 3. Experiment Validations and Result Discussions

In this section, the experimental data and processing strategy are first presented. Then, the performance of the tightly integrated PPP−B2b/INS and PPP−B2b/INS using the MEMS IMU is evaluated.

### 3.1. Experiment Data and Processing Strategy

**Figure 2:**
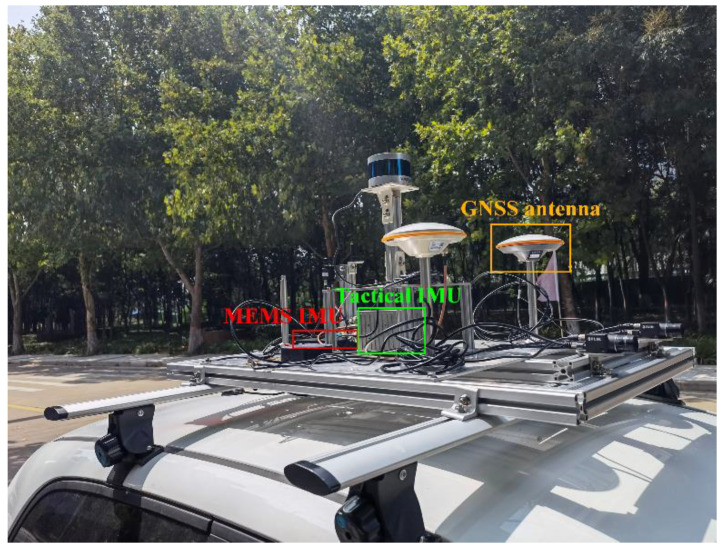
Vehicle−mounted platform of the experiment.

**Figure 3:**
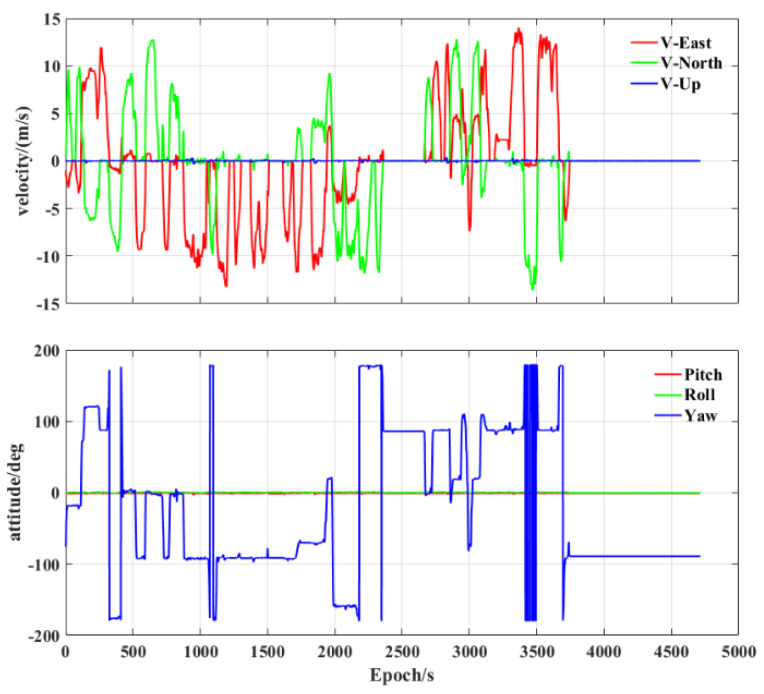
Vehicle motion status of the experiment.

To assess the performance of the tightly integrated PPP−B2b/INS, we conducted a vehicle experiment in an urban environment in Zhengzhou, China. The experimental platform is presented in Figure 2. The experiment was carried out on 18 October 2022, with a total duration of about 1.5 h. A stationary period at the beginning of the experiment was used for the initial alignment of the integrated navigation system. During the experiment, the vehicle performed many maneuvers, such as remaining stationary, accelerating and decelerating, and turning. Its velocity and attitude changes are shown in Figure 3, where the initial stationary period is not included.

**Figure 4:**
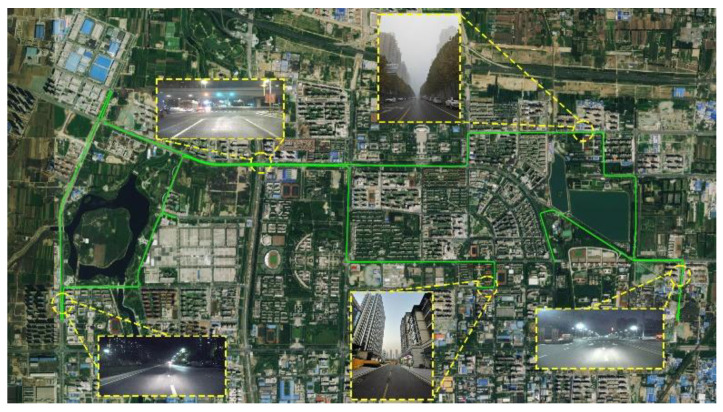
The trajectory of the experiment.

**Figure 5:**
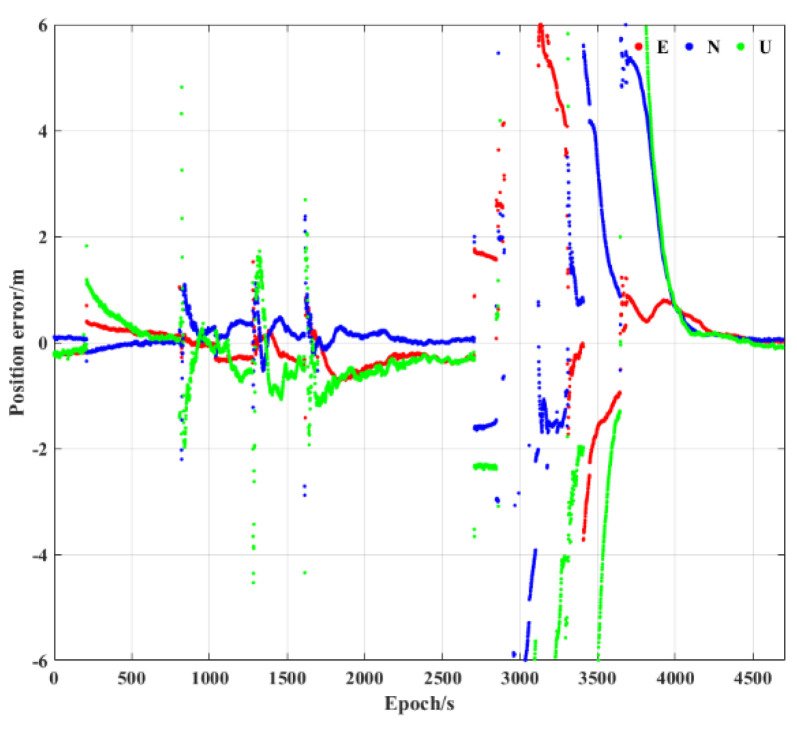
The position error of PPP in the experiment.

The trajectory of the vehicle is shown in Figure 4, wherein some challenging experimental scenes are also shown. The position error of PPP is shown in Figure 5. The GNSS signals are mainly blocked by tall residential buildings, trees, and viaducts during the experiment, leading to large multi−path errors or satellite signal loss, causing errors of several meters or positioning interruptions in PPP and PPP/INS loosely coupled integration. Only the performance of the tightly integrated PPP/INS is discussed below.

The SinoGNSS K803 card was used to receive the RT corrections of the BDS−3 PPP−B2b during the experiment, and BNC software version 2.12 was used to receive the RT SSR corrections broadcasted by GFZ through ntrip.gnsslab.cn operated by Wuhan University, China. The vehicle used in the experiment was armed with an additional GNSS antenna (CHCNAV AT312), a receiver card unicorecomm UB4B0, a tactical IMU SNC300A−DGI, and a MEMS IMU ADIS−16470. Raw observations of the GNSS were recorded at 1 Hz, while those of the MEMS IMU and tactical IMU were recorded at 100 Hz and 200 Hz, respectively. The above two IMU precision specifications are shown in Table 1.

In the experiment, a reference station was set up nearby in an open environment, and a smooth solution of the tightly coupled multi−GNSS RTK/INS conducted by the commercial software Inertial Explorer (IE) 8.9 was utilized for reference. The time alignment of integration navigation was maintained with GPST, and the offset between the GNSS antenna and the IMU center was measured early to implement the spatial alignment. The processing strategies are shown in Table 2.

### 3.2. Performance of Tightly Integrated PPP−B2b/INS

**Figure 6:**
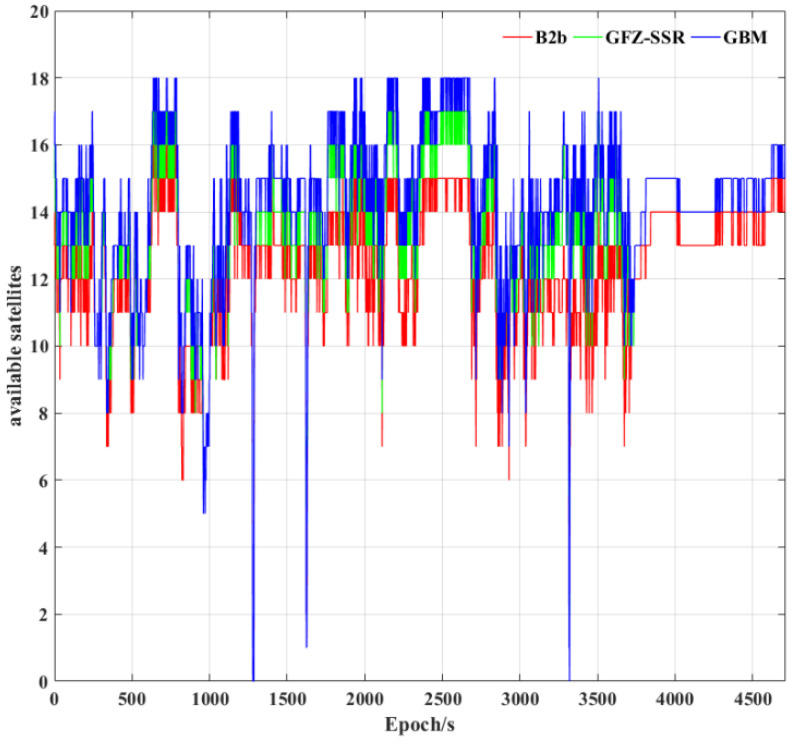
The available satellites for BDS−3 and GPS during the experiment.

In this section, the performance of the tightly integrated BDS−3 PPP−B2b/INS in the urban environment is investigated using the tactical IMU. To analyze the accuracy of the positioning, velocimetry, and attitude determination, it is compared with the PPP/INS tight integration using the GFZ broadcast RT corrections (referred to as GFZ−SSR) and the GFZ post precise product (referred to as GBM). The number of available satellites of the BDS−3 and GPS using the three precise products during the experiments is shown in Figure 6. It should be mentioned that the number of satellites available in this paper represents the number of visible satellites which have precise orbits and clock offsets. It can be seen from Figure 6 that GBM has the maximum number of available satellites. Table 3 further provides the statistics on the average available satellites using the three precise products. The average number of available satellites is 12.34 when PPP−B2b corrections were used, the corresponding results for GFZ−SSR and GBM were 13.64 and 14.29, respectively. Currently, the number of satellites supported by the PPP−B2b service is still relatively low due to the limitation of the number and distribution of ground monitoring stations, which may affect its positioning performance. In addition, the post−precise products support more satellites and a higher product accuracy due to a better processing strategy and more observation stations around the world.

**Figure 7:**
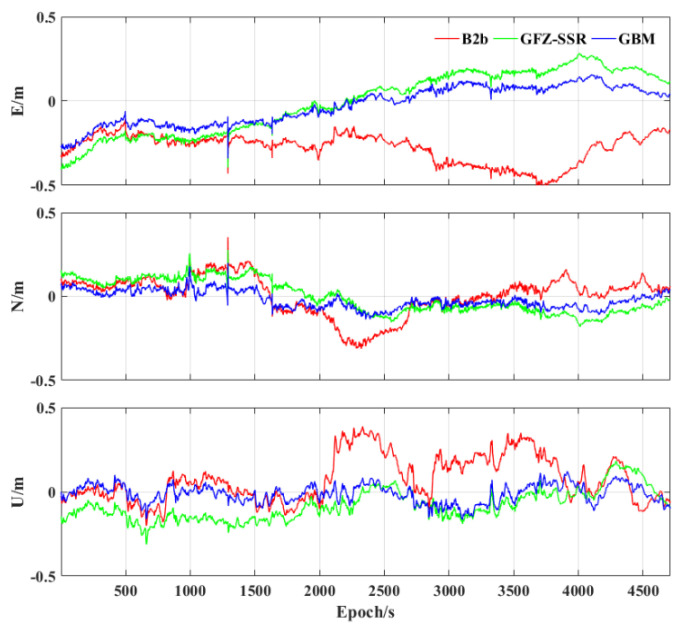
Positioning error of the three precise products using tactical IMU.

First, Figure 7 shows the positioning error using different precise products. It can be seen that all three precise products used have the largest positioning error in the E component, which may be related to the GNSS observation conditions of the experiment. Three GNSS short interruptions happened in the experiment. These three solutions all can provide continuous, reliable, and precise positioning during the GNSS short interruption. There is still an obvious difference in the positioning accuracy between the two RT products and the post−precise products. Decimeter−level positioning precision can be achieved using these three products. Table 4 further presents the statistical root mean square (RMS) error of the three precise products. The positioning accuracies in the E, N, and U components using PPP−B2b corrections are 0.292, 0.115, and 0.155 m, respectively, while they are 0.183, 0.098, and 0.122 m, respectively, using GFZ−SSR and 0.115, 0.055, and 0.056 m, respectively, using GFZ post−precise products. Compared with GFZ RT products, the 3D positioning accuracy of the BDS−3 PPP−B2b still has a gap of about 1 dm and 2 dm compared with GFZ post−precise products.

**Figure 8:**
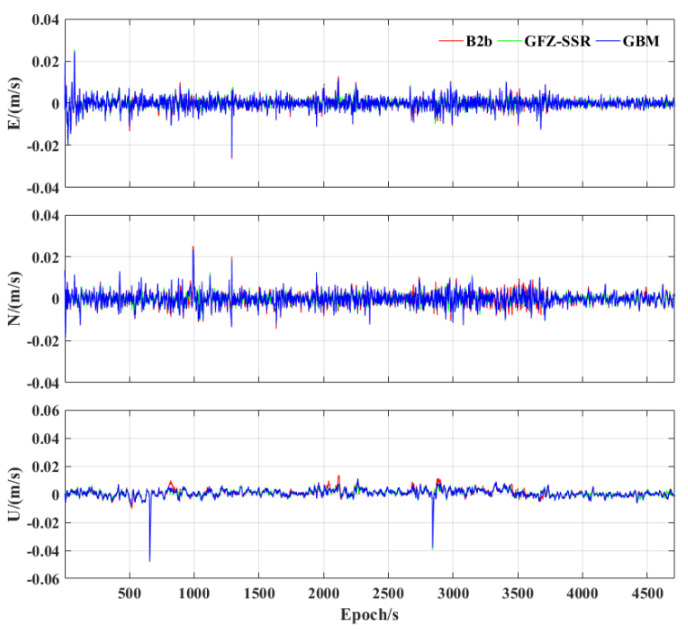
Velocity error of the three precise products using tactical IMU.

Regarding velocimetry, the velocity error using different products is shown in Figure 8. It can be seen from Figure 8 that the accuracy of the velocimetry is very stable, and there is no significant difference in the velocimetry error using different precise products. Table 5 depicts the RMS statistics in the E, N, and U components using different products. The accuracy of the velocimetry using the three precise products in the E, N, and U components is about 0.3 cm/s. The accuracy of the velocimetry with GFZ−SSR is slightly better compared to the GFZ post−precise products, probably due to its higher satellite orbit and clock offset sampling rate.

**Figure 9:**
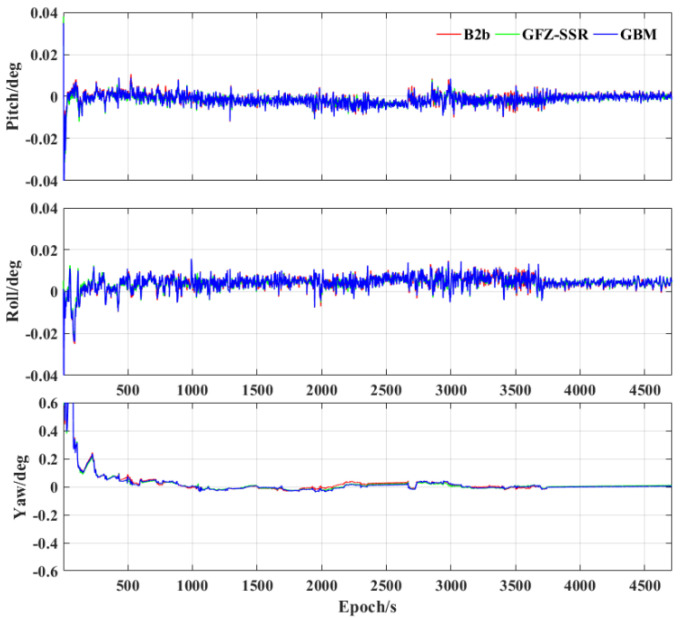
Attitude error of the three precise products using tactical IMU.

Finally, Figure 9 shows the attitude error of the pitch, roll, and yaw using different precise products. It can be seen that there is no significant difference in attitude errors using different precise products. In addition, the accuracy of the yaw is significantly worse than that of the pitch and roll. The accuracy of the yaw at the beginning of the experiment is significantly lower than that at other periods due to the weak observability and the absence of obvious movement of the vehicle [38]. Table 6 further shows the attitude error RMS statistics using the three precise products. The accuracy of the pitch and roll with three precise products is all better than 0.01 deg, and the yaw accuracy is about 0.1 deg. As with the accuracy of the velocimetry, the attitude accuracy using the three precise products also has no significant differences. Hence, the accuracy of the velocimetry and attitude determination mainly depends on the precision of the IMU in the PPP/INS tight integration, and PPP−B2b/INS can also obtain an equally accurate velocity and attitude as the scheme using post−precise products.

### 3.3. Tightly Integrated PPP−B2b/INS with Low−Cost MEMS IMU

The capabilities of the BDS−3 PPP−B2b/INS with a tactical IMU were evaluated in the previous section. To perform a further analysis of the MEMS IMU for wide applications, this section compared the performance of the tactical IMU and the MEMS IMU using the BDS−3 PPP−B2b service and GFZ post products.

**Figure 10:**
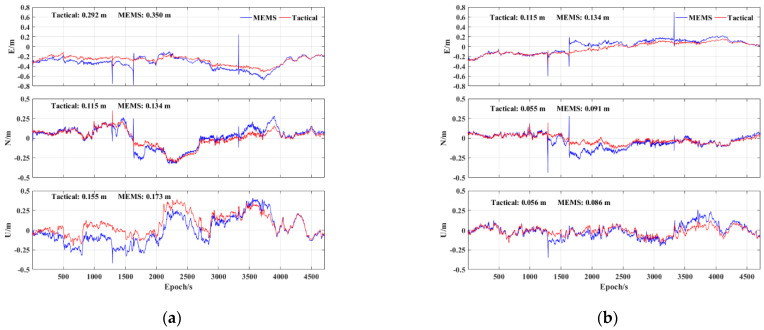
PPP/INS tight integration positioning error of tactical IMU and MEMS IMU: (**a**) BDS−3 PPP−B2b RT corrections; (**b**) GFZ post−precise products.

**Figure 11:**
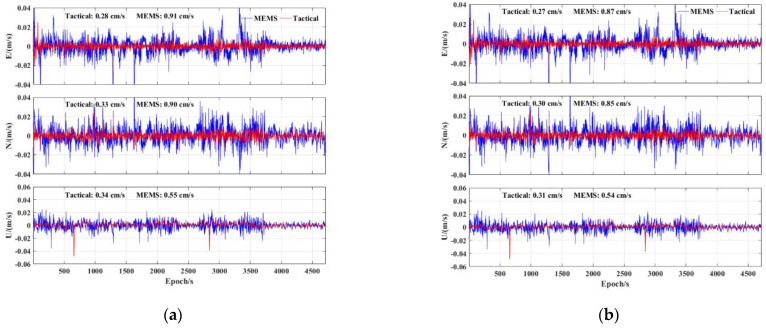
PPP/INS tight integration velocimetry error of tactical IMU and MEMS IMU: (**a**) BDS−3 PPP−B2b RT corrections; (**b**) GFZ post−precise products.

First, Figure 10 shows the positioning error of different IMUs using the PPP−B2b RT corrections and GFZ post−precise products. It can be seen that the positioning performance of the tactical IMU and MEMS IMU was significantly different, especially during GNSS signal interruption. During short GNSS interruptions, the positioning error using the MEMS IMU accumulated rapidly, while the tactical IMU still maintained a better positioning accuracy. The RMS statistics of the positioning error with different IMUs using the two precise products are also shown in Figure 11. The positioning accuracies in the E, N, and U components of the PPP−B2b/MEMS INS tight integration are 0.350, 0.134, and 0.173 m, respectively, while they were improved by 16.6%, 14.2%, and 10.4%, respectively, using the tactical IMU, and 14.2%, 39.6%, and 34.9%, respectively, using the tactical IMU and post−precise products.

Then, regarding the velocimetry, Figure 11 shows the velocity error of the two precise products using different IMUs. Compared with the MEMS IMU, the velocimetry accuracy of the tactical IMU was significantly improved. The velocimetry accuracy of the MEMS IMU is better than 1 cm/s in all the E, N, and U components.

**Figure 12:**
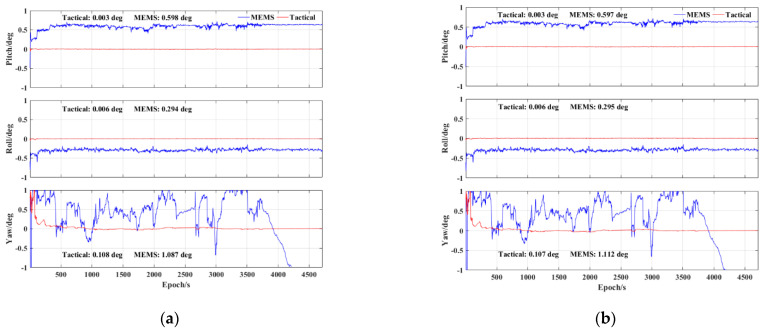
PPP/INS tight integration attitude error of tactical IMU and MEMS IMU: (**a**) BDS−3 PPP−B2b RT corrections; (**b**) GFZ post−precise products.

**Figure 13:**
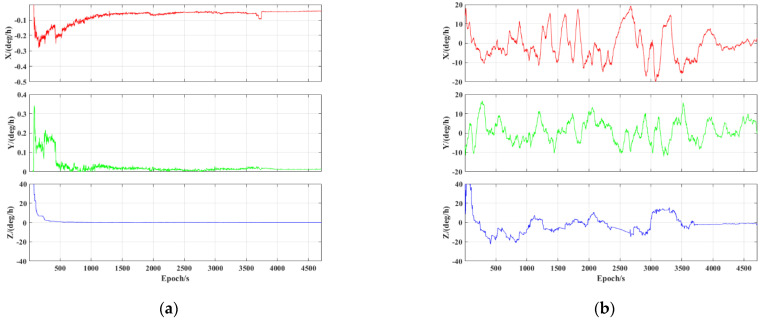
The gyro biases estimation errors of the two IMUs: (**a**) tactical IMU; (**b**) MEMS IMU.

Finally, Figure 12 presents the corresponding attitude errors. It can be seen that the accuracy of the pitch, roll, and yaw using the MEMS IMU has decreased significantly compared to the tactical IMU. This is mainly due to the inherent poor accuracy of the MEMS IMU and the fact that the biases estimation accuracy of the MEMS IMU is not as accurate as that of the tactical IMU. The gyro biases estimation errors of the two IMUs are shown in Figure 13. In addition, the yaw error presented a rapid increase in the final vehicle stationary period using the MEMS IMU. The accuracy of the pitch, roll, and yaw were about 0.6, 0.3, and 1.1 deg, respectively, using the MEMS IMU. Both the results in Figure 11 and Figure 12 indicate that the accuracy of the velocimetry and attitude determination in the PPP/INS tight integration mainly depends on the precision of the IMU.

## 4. Conclusions

PPP−B2b supports RT−PPP as a featured service of BDS−3. In contrast to RTS initiated by IGS, PPP−B2b does not require internet communication. RT−PPP based on satellite communication has more advantages, especially for areas without internet communication coverage or those with a congested network and a poor network signal. However, satellite signals are easily blocked within a complicated environment. To provide a continuous and secure position during GNSS signal blocking and interruption, this paper proposes a tightly integrated BDS−3 PPP−B2b/INS model and analyzes its performance in the urban environment, and is also compared with GFZ RT corrections and post−precise products. In addition, the performance of the tightly integrated PPP−B2b/INS with the MEMS IMU for mass applications is also evaluated.

Currently, the PPP−B2b service only supports BDS−3 and GPS, and the number of supported satellites is still limited. The average number of available satellites in this experiment is 12.34, which still is slightly lower compared with GFZ RT precise products. The experiment results show that the tightly integrated PPP−B2b/INS can provide continuous and reliable positioning during short interruptions in GNSS signals. The positioning accuracies in the E, N, and U components of the PPP−B2b/INS tight integration are 0.292, 0.115, and 0.155 m, respectively. Hence, PPP−B2b/INS tight integration can achieve a decimeter−level positioning accuracy in the urban environment and meet the positioning requirements of certain scenarios. Compared with the performance using GFZ RT products, the 3D positioning accuracy of the PPP−B2b/INS still has a gap of about 1 dm and 2 dm compared to when GFZ post−precise products are utilized. Additionally, the accuracy of the velocimetry and attitude determination mainly depends on the precision of the IMU in the PPP/INS tight integration, since no significant difference is found between RT and post products. These three solutions can all achieve a good accuracy using the tactical IMU; the velocimetry accuracy of the PPP−B2b/INS is about 0.3 cm/s in all the E, N, and U components, and the accuracy of the pitch and roll is better than 0.01 deg, while that of the yaw is about 0.1 deg. When the low−cost MEMS IMU is used, its performance degraded significantly, in which the attitude accuracy decreased most significantly.

With the development of BDS−3, the PPP−B2b service will quickly support more satellites and more satellite systems, and the precision of the PPP−B2b orbit and clock offset corrections will be improved, resulting in a better performance of the tightly integrated PPP−B2b/INS. In addition, the INS error will accumulate rapidly when the GNSS signals are interrupted for a long time; hence, the performance of the PPP/INS will degrade quickly. Therefore, other sensors such as an odometer and camera can be further integrated to improve the performance. Additionally, integrity monitoring of PPP/INS should be considered in the future.

## Figures and Tables

**Table 1:** Precision specifications of the above IMUs utilized in the experiment.

IMU	Sampling Rates	Gyro Bias (deg/h)	Accelerometer Bias (mGal)	$\mathrm{Angular}\;\mathrm{Random}\;\mathrm{Walk}\;(\deg/\sqrt{h})$
SNC300A−DGI	200 Hz	0.3	50	0.05
ADIS−16470	100 Hz	8	1300	0.34

**Table 2:** Processing strategy of PPP−B2b/INS.

Parameter	Processing Strategy
Satellite systems	BDS−3; GPS
Frequency	BDS−3 (C): B1I + B3I; GPS (G): L1 + L2
Sampling rate	1 Hz
Satellite elevation cut−off angle	7°
Receiver clock offsets	Modeled as white noise
Tropospheric delay	Using the Saastamoinen model where the residual component is modeled as a random walk process [35]
Satellite and receiver antenna phase center offset	igs14.atx
Weight for observations	Elevation−dependent weight [36]
Inter−system bias	Modeled as a random walk process
Biases of accelerometer and gyro	Modeled as a random walk process
Quality control strategy	Detection, identification, and adaptation (DIA) [37]

**Table 3:** Average number of available satellites.

Scheme	Available Satellites
B2b	12.34
GFZ−SSR	13.64
GBM	14.29

**Table 4:** RMS statistics of positioning error for the three precise products using tactical IMU (unit: m).

Scheme	E	N	U	H	3D
B2b	0.292	0.115	0.155	0.314	0.350
GFZ−SSR	0.183	0.098	0.122	0.208	0.241
GBM	0.115	0.055	0.056	0.127	0.139

**Table 5:** RMS statistics of velocity error for the three precise products using tactical IMU (unit: cm/s).

Scheme	E	N	U
B2b	0.28	0.33	0.34
GFZ−SSR	0.25	0.29	0.30
GBM	0.27	0.30	0.31

**Table 6:** RMS statistics of attitude error for the three precise products using tactical IMU (unit: deg).

Scheme	Pitch	Roll	Yaw
B2b	0.003	0.006	0.108
GFZ−SSR	0.003	0.005	0.107
GBM	0.003	0.006	0.107

## Data Availability

The datasets used in this study are managed by the Institute of Surveying and Mapping, Information Engineering University, Zhengzhou, China, and are available on request from the corresponding author.
